# Early Detection of Prescription Drug Abuse Using Doctor Shopping Monitoring From Claims Databases: Illustration From the Experience of the French Addictovigilance Network

**DOI:** 10.3389/fpsyt.2021.640120

**Published:** 2021-05-17

**Authors:** Thomas Soeiro, Clémence Lacroix, Vincent Pradel, Maryse Lapeyre-Mestre, Joëlle Micallef

**Affiliations:** ^1^Aix-Marseille Université, Inserm, UMR 1106, Hôpitaux Universitaires de Marseille, Service de Pharmacologie Clinique, Centre d'évaluation et d'information sur la Pharmacodépendance – Addictovigilance, Marseille, France; ^2^Université Paul Sabatier, Inserm, CIC 1436, Centre Hospitalier Universitaire de Toulouse, Service de Pharmacologie Clinique, Centre d'évaluation et d'information sur la Pharmacodépendance – Addictovigilance, Toulouse, France

**Keywords:** doctor shopping, prescription drug abuse, claims database, signals detection, addictovigilance, opioids, benzodiazepines, methylphenidate

## Abstract

Opioid analgesics and maintenance treatments, benzodiazepines and z-drugs, and other sedatives and stimulants are increasingly being abused to induce psychoactive effects or alter the effects of other drugs, eventually leading to dependence. Awareness of prescription drug abuse has been increasing in the last two decades, and organizations such as the International Narcotics Control Board has predicted that, worldwide, prescription drug abuse may exceed the use of illicit drugs. Assessment of prescription drug abuse tackles an issue that is hidden by nature, which therefore requires a specific monitoring. The current best practice is to use multiple detection systems to assess prescription drug abuse by various populations in a timely, sensitive, and specific manner. In the early 2000's, we designed a method to detect and quantify doctor shopping for prescription drugs from the French National Health Data System, which is one of the world's largest claims database, and a first-class data source for pharmacoepidemiological studies. Doctor shopping is a well-known behavior that involves overlapping prescriptions from multiple prescribers for the same drug, to obtain higher doses than those prescribed by each prescriber on an individual basis. In addition, doctor shopping may play an important role in supplying the black market. The paper aims to review how doctor shopping monitoring can improve the early detection of prescription drug abuse within a multidimensional monitoring. The paper provides an in-depth overview of two decades of development and validation of the method as a complementary component of the multidimensional monitoring conducted by the French Addictovigilance Network. The process accounted for the relevant determinants of prescription drug abuse, such as pharmacological data (e.g., formulations and doses), chronological and geographical data (e.g., impact of measures and comparison between regions), and epidemiological and outcome data (e.g., profiles of patients and trajectories of care) for several pharmacological classes (e.g., opioids, benzodiazepines, antidepressants, and methylphenidate).

## Introduction

Opioid analgesics and maintenance treatments, benzodiazepines and z-drugs, and other sedatives and stimulants are increasingly being abused to induce psychoactive effects or alter the effects of other drugs, eventually leading to dependence (1). Awareness of prescription drug abuse has been increasing in the last two decades, and organizations such as the International Narcotics Control Board has predicted that, worldwide, prescription drug abuse may exceed the use of illicit drugs (2). Prescription drug abuse is now qualified as an epidemic in economically developed countries, particularly in North America (1, 3, 4).

Many studies pointed out an increasing trend of prescription drug abuse across European countries, highlighting the need for a specific monitoring (5–9). Several factors may explain this trend, such as a greater ease in obtaining prescription drugs than illicit drugs, a lower risk of arrest for trafficking, a higher social acceptability of their abuse, their higher purity, and their more predictable doses (6).

Assessment of prescription drug abuse tackles an issue that is hidden by nature, which therefore requires a specific monitoring. A single data source is rarely enough to assess such a complex phenomenon (10). The current best practice is to use multiple detection systems to assess prescription drug abuse by various populations in a timely, sensitive, and specific manner (11). By using various tools to mine epidemiological data, assess the pharmacological properties of the drugs, and assess the social contexts where the drugs are used, these systems demonstrated their usefulness to detect emerging trends earlier and intervene more quickly to protect the public from associated risks (12). Among these tools, assessing doctor shopping through overlapping prescriptions, multiple prescribers, or pharmacy shopping was implemented in several countries (13–19). Therefore, the paper aims to review how doctor shopping monitoring can improve the early detection of prescription drug abuse within a multidimensional monitoring. The paper provides an in-depth overview of two decades of development and validation of the method as a complementary component of the multidimensional monitoring conducted by the French Addictovigilance Network.

## Relevance of Doctor Shopping As a Proxy for Prescription Drug Abuse

Drug-abusing patients may develop drug-seeking behavior to meet their need. Among them, doctor shopping has long been described, in several countries (e.g., North America, Europe, Asia, and Oceania) and for several pharmacological classes (e.g., opioids, stimulants, and benzodiazepines) (13–19). Doctor shopping involves overlapping prescriptions from multiple prescribers for the same drug, to obtain higher doses than those prescribed by each prescriber on an individual basis. Doctor shopping is based on circumventing the optimal one-to-one patient-prescriber relationship, and therefore on a lack of medical management, because one given prescriber does not know that other prescribers are also prescribing the same drug. The lack of medical management in addition to high doses increase the risks for adverse outcomes, such as high-risk use, overdose, and death (13, 20–24).

Among many diverted means for obtaining prescription drugs (e.g., friends or relatives, black market, or internet), doctor shopping is reported as one of the most frequent ones (25–27). In addition, obtaining prescription drugs from a dealer raises the question of how dealers obtain the prescription drugs they sell (28). Although the question is difficult to answer with a strong evidence, field studies suggest that doctor shopping may play an important role in supplying the black market (29–32). Notably, without regard to the final consumer (i.e., whether the patient himself or a subsequent purchaser), the concern for the lack of medical management remains, along with the risks associated with it.

## How to Quantify Doctor Shopping?

Doctor shopping is difficult to monitor because the patient often attempts to hide the abuse and the prescribers may not even realize that they have been deceived. These observations underline the limitations of interviewing the prescribers or patients, and therefore, highlight the added value of claims databases to quantify doctor shopping objectively. Several teams from different countries have developed methods to detect doctor shopping in claims databases (13–19). The methods face two main challenges: a proper design of the method to accurately detect drug-abusing patients and the use of a data source that is representative of the population of interest.

### First Challenge: The Design of the Method

The method must be both specific (i.e., must not red flag non-abusing patients) and sensible (i.e., must not miss real drug-abusing patients). Nevertheless, there is no standard definition of doctor shopping, and therefore, no gold standard method. Most studies assessing doctor shopping rely on the number of prescribers or pharmacies visited, without regard to successive and overlapping prescriptions (33). Such methods may overestimate abuse, because successive prescriptions from different prescribers may be legitimately needed, particularly in cancer and palliative care (34), or in similar situations when a general practitioner refers a regular patient to a specialist (35). Other situations involving successive prescribers may not be related to abuse or restricted to psychoactive prescription drugs, but rather related to prescriber factors (e.g., inconvenient hours or locations, long waiting times, or personal characteristics of the prescriber), illness factors (e.g., persistence of symptoms, lack of understanding, or lack of confidence in diagnosis or treatment), or psychological factors (e.g., anxiety leading to dose stockpiling) (36, 37).

Conversely, overlapping prescription is at the core of the safety concern, because it is the reason for the lack of medical management. Interestingly, a study compared the diagnostic odds ratios for opioid overdose of nine definitions of pharmacy shopping, using a multistate Medicaid claims database in the USA (38). The overdose rate was higher in patients with overlapping prescriptions than in patients with only pharmacy shopping. In addition, another study quantified episodes of multiple prescriber for benzodiazepines using a two-year cohort in Japan. Consecutive overlapping prescriptions had the best accuracy to detect patients with potentially questionable prescribed quantities, and predict patients with episodes of multiple prescriber in the subsequent year (19).

**Figure 1:**
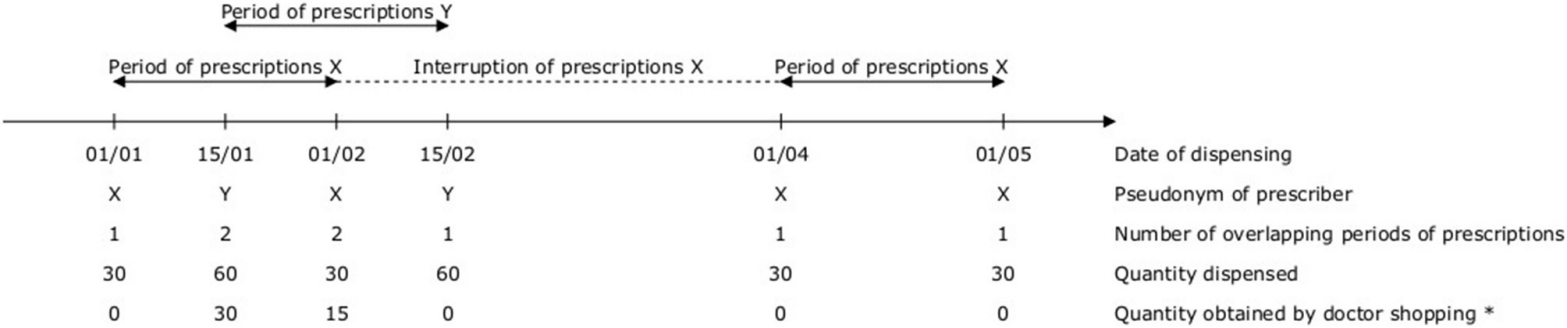
Method to detect and quantify doctor shopping for prescription drugs, accounting for overlapping prescriptions. *The quantity obtained by doctor shopping is calculated as Qd–Qd/n, where Qd is the quantity dispensed, n is the number of overlapping periods of prescriptions, and Qd/n is the quantity that would have been dispensed with only one prescriber.

**Figure 2:**
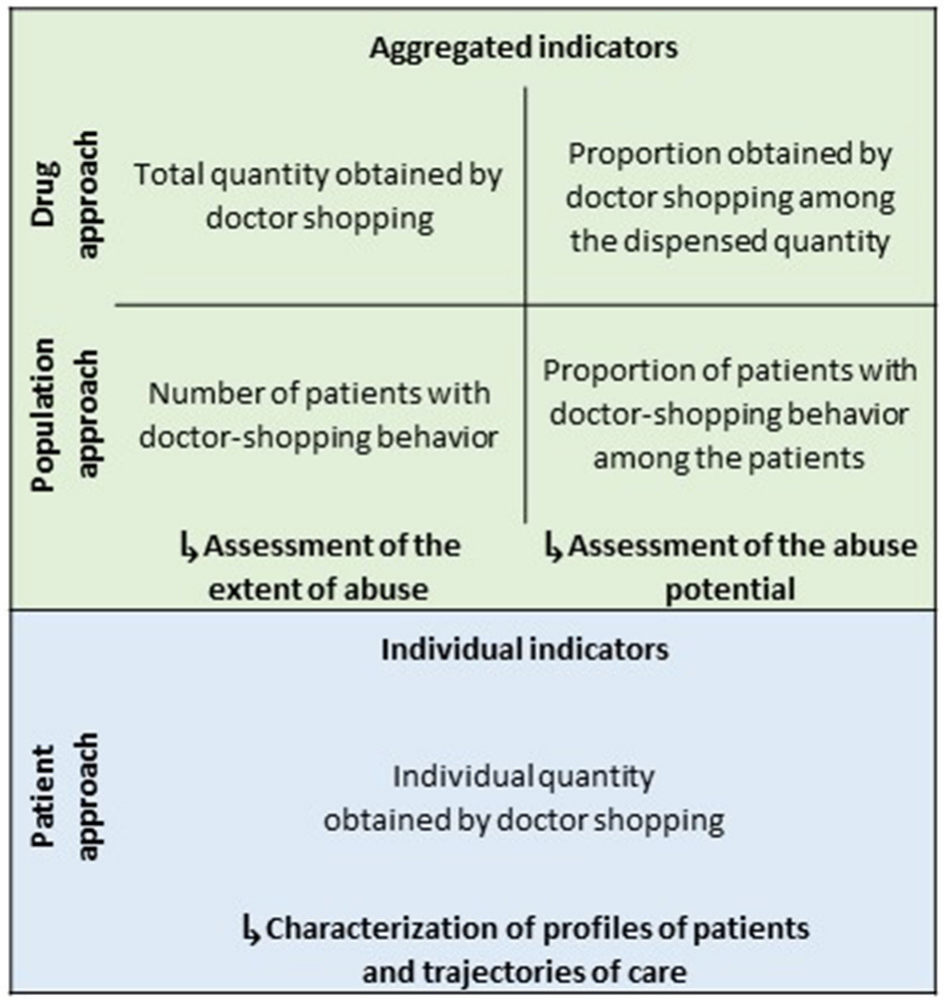
Complementary indicators provided by the method.

In the early 2000's, we designed a method to detect and quantify doctor shopping for prescription drugs, accounting for overlapping prescriptions (14, 39–45) (Figure 1). To detect overlapping prescriptions, the method relies on periods of prescriptions, defined as the period between the first and last dispensing for each prescriber of each patient (i.e., the period during which a patient consults a prescriber). If there is a longer delay than a predefined threshold between two consecutive dispensings, the period of prescriptions is interrupted. During an interruption, a prescription from another prescriber is not considered as overlapping to avoid the overestimation of doctor shopping. If there are overlapping periods of prescriptions, there is a lack of medical management, and a share of the drug prescribed is considered to be obtained by doctor shopping. The method provides aggregated drug-level indicators (e.g., total quantity and proportion obtained by doctor shopping) and population-level indicators (e.g., number and proportion of patients with doctor shopping behavior), and individual patient-level indicators (e.g., individual quantity obtained by doctor shopping) (Figure 2). Taken together, these complementary indicators enable to assess the extent of abuse and abuse potential of prescription drugs, and characterize profiles of patients with doctor shopping behavior and their trajectories of care.

Notably, the method deliberately relies on a strict design to specifically detect overlapping prescriptions rather than the number of prescribers or pharmacies visited. In addition, the quantity obtained by doctor shopping is not the entire quantity received by a patient with doctor shopping behavior, but only the quantity received in addition to what is dispensed with only one prescriber. The underlying reason for this design is that the patient may legitimately need the drug for a medical use at the quantity prescribed by one prescriber. This design helps to rule out the hypothesis of pseudoaddiction [i.e., doctor shopping driven by insufficient dosing (46)], because it enables to discriminate patients who receive high doses in addition to a treatment considered legitimate (14, 45).

### Second Challenge: A Representative Data Source

To enable an accurate quantification of doctor shopping, the database must be representative of the population of interest. In addition, the database must identify each health professional and health care consumer by a consistent pseudonym over time and across geography. Given that claims databases were initially designed for medicoadministrative purposes, it is far from trivial in practice. For example, in the USA, health insurance plans only cover residents by states or focus on a specific subset of the population (e.g., Medicaid covers low-income populations, while private insurances are employment-based). Notably, the use of a non-representative population may bias the results, because socioeconomic status is associated with abuse (47–49). In addition, some regulation [e.g., the 42 CFR part 2 in the USA, which aims to ensure confidentiality of records from federally funded drug and alcohol treatment centers (50)] may further complicate the use of claims databases.

In this regard, the French National Health Data System is a first-class data source for pharmacoepidemiological studies, as one of the world's largest claims database, whose representativeness is almost perfect (51–53). The National Health Data System prospectively merges pseudonymized records of claims from all the French health insurance plans, the national hospital-discharge database, and the national death registry (54). Because the coverage by a health insurance plan is mandatory in France, the French National Health Data System covers almost 100% of the 67 million inhabitants, from birth to death, independently of the socioeconomic status and region of residence. In addition, each health professional and health care consumer is identified by a consistent pseudonym over time and across geography. As a result, the French National Health Data System enables a nationwide and exhaustive quantification of doctor shopping.

In the last two decades, the French National Health Data System has been extensively used for pharmacoepidemiological research, including some large-scale studies that have led to major public health interventions (55, 56). Among them, many studies have focused on psychoactive prescription drugs (57–63).

## Validation of Doctor Shopping As a Pharmacological Tool

Before using doctor shopping as a proxy for prescription drug abuse, there is a need for an in-depth customized validation process within the health system of interest. The lack of a gold standard method makes a classical statistical validation process impossible (i.e., sensitivity, specificity, and predictive values). Therefore, an empirical approach is required to assess the external validity of the proxy in a given health system for several pharmacological classes. Such a process should rely on linking doctor shopping to relevant determinants of prescription drug abuse, such as pharmacological data (e.g., formulations and doses), chronological and geographical data (e.g., impact of measures and comparison between regions), and epidemiological and outcome data (e.g., profiles of patients and trajectories of care).

In the last two decades, we have conducted such an empirical validation of our method (14, 39–45) (Table 1). The process has provided solid evidence that the method is a relevant proxy for prescription drug abuse within the French health system, because it has always demonstrated an excellent external validity. In particular, the method demonstrated to be a useful pharmacological tool, able to provide detailed results by discriminating drugs, formulations, and doses.

**Table 1:** Empirical validation of the method accounting for overlapping prescription, in the last two decades, in France.

**References**	**Date**	**Setting**	**Prescription drugs under study**	**Number of patients included**	**Main findings**
Pradel et al. (14)	1999 and 2000	Two million inhabitants in South East France	Buprenorphine maintenance treatment	3,259	225,351 DDD were obtained by doctor shopping, corresponding to 18.7% of the quantity dispensed. Doctor shopping was highly concentrated on a minority of patients (i.e., 8.5% of patients accounted for 45.4% of the quantity obtained by doctor shopping).
Pradel et al. (39)	2000 to 2005	Two million inhabitants in South East France	Buprenorphine maintenance treatment	>2,600 each semester	Doctor shopping increased from 2000 (i.e., 14.9% of the quantity dispensed) to 2004 (i.e., 21.7% of the quantity dispensed), and decreased in 2005 (i.e., 16.9% of the quantity dispensed) following the implementation of a prescription monitoring program. The number of patients remained stable from 2000 to 2005.
Pradel et al. (40)	2003	One million inhabitants in South West France	Benzodiazepines	128,230	Benzodiazepines were ranked according to their abuse potential in real-life setting. The proportion obtained by doctor shopping was the highest for flunitrazepam 1 mg (i.e., 42.8% of the quantity dispensed), then for diazepam 10 mg (i.e., 3.2% of the quantity dispensed), and clorazepate 50 mg (i.e., 2.7% of the quantity dispensed).
Rouby et al. (41)	2005	Five million inhabitants in South East France	Antidepressants and benzodiazepines as comparator	410,525	Tianeptine ranked first among antidepressants for the proportion obtained by doctor shopping (i.e., 2.0% of the quantity dispensed), and was close to benzodiazepines with a well-known abuse potential in real-life setting.
Nordmann et al. (42)	2008	14 million inhabitants in three regions in South France (i.e., Provence-Alpes-Côte d'Azur, Rhône-Alpes, and Midi-Pyrénées)	Opioids	885,941 in Provence-Alpes-Côte d'Azur 945,102 in Rhône-Alpes 386,834 in Midi-Pyrénées	The quantity obtained by doctor shopping in Provence-Alpes-Côte d'Azur (i.e., 213 DDD/1,000 inhabitants) was two-fold higher than in Rhône-Alpes (i.e., 115 DDD/1,000 inhabitants) and in Midi-Pyrénées (i.e., 106 DDD/1,000 inhabitants). A signal emerged for oxycodone in Midi-Pyrénées.
Ponté et al. (43)	2013	14 million inhabitants in South France	Opioids and benzodiazepines as comparator	1,257,246	The proportion obtained by doctor shopping was the highest for the highest doses of morphine (i.e., 8.4% of the quantity dispensed for morphine 200 mg) and oxycodone (i.e., 2.8% of the quantity dispensed for oxycodone 80 mg), and for nasal and transmucosal fentanyl (i.e., respectively 4.1 and 3.3% of the quantity dispensed).
Soeiro et al. (44)	2010 and 2016	67 million inhabitants in France	Oxycodone	67,838 in 2010 212,753 in 2016	There was a three-fold increase in doctor shopping in line with population exposure. The quantity obtained by doctor shopping increased with the dose for both immediate-release and extended-release tablets.
Soeiro et al. (45)	2016	67 million inhabitants in France	Methylphenidate	63,739	Patients with heavy doctor shopping behavior were older, received more concomitant dispensing of antipsychotics and opioid maintenance treatments, and had more prescribers.

*DDD, defined daily dose*.

### Detecting Prescription Drugs With a High Abuse Potential

The method was first developed for buprenorphine maintenance treatment (14, 39), which was expected to have a high abuse potential in the real-life setting. In France, a wide access to maintenance treatments is ensured by an office-based setting for the majority of patients (64, 65). In parallel of a marked decrease in lethal heroin overdoses, a concern emerged along with observations of abuse (e.g., injection of crushed tablets, snorting, association with benzodiazepines such as flunitrazepam, and deaths) and an increasing buprenorphine black market (14). Interestingly, evidence of multiple prescribers for buprenorphine maintenance treatment was also described, but without quantifying the buprenorphine maintenance treatment involved, nor accounting for overlapping prescriptions (66).

A study was conducted among the 3,259 patients who received buprenorphine maintenance treatment in a population of two million inhabitants in South East France in 1999 and 2000. The method found that 225,351 defined daily doses (DDD) were obtained by doctor shopping, corresponding to 18.7% of the quantity dispensed (14). Doctor shopping was highly concentrated on a minority of patients (i.e., 8.5% of patients accounted for 45.4% of the quantity obtained by doctor shopping).

As a result, the health insurance implemented a prescription monitoring program for opioid maintenance therapies in 2004, for both public health and economic concerns. Patients who received >32 mg/day of buprenorphine maintenance treatment (i.e., twice the maximum recommended dose) were proposed a contract of care, including the choice of a single prescriber and pharmacist for buprenorphine maintenance treatment. Patients with particularly high doses who did not respond to the convocation, or did not respect their contract of care, could be prosecuted, or excluded from the health insurance plan. A second assessment of doctor shopping from 2000 to 2005 in the same population found that the prescription monitoring program led to a decrease in doctor shopping, without decreasing the access to buprenorphine maintenance treatment (39).

### Ranking Prescription Drugs Within a Pharmacological Class Known for Abuse

**Figure 3:**
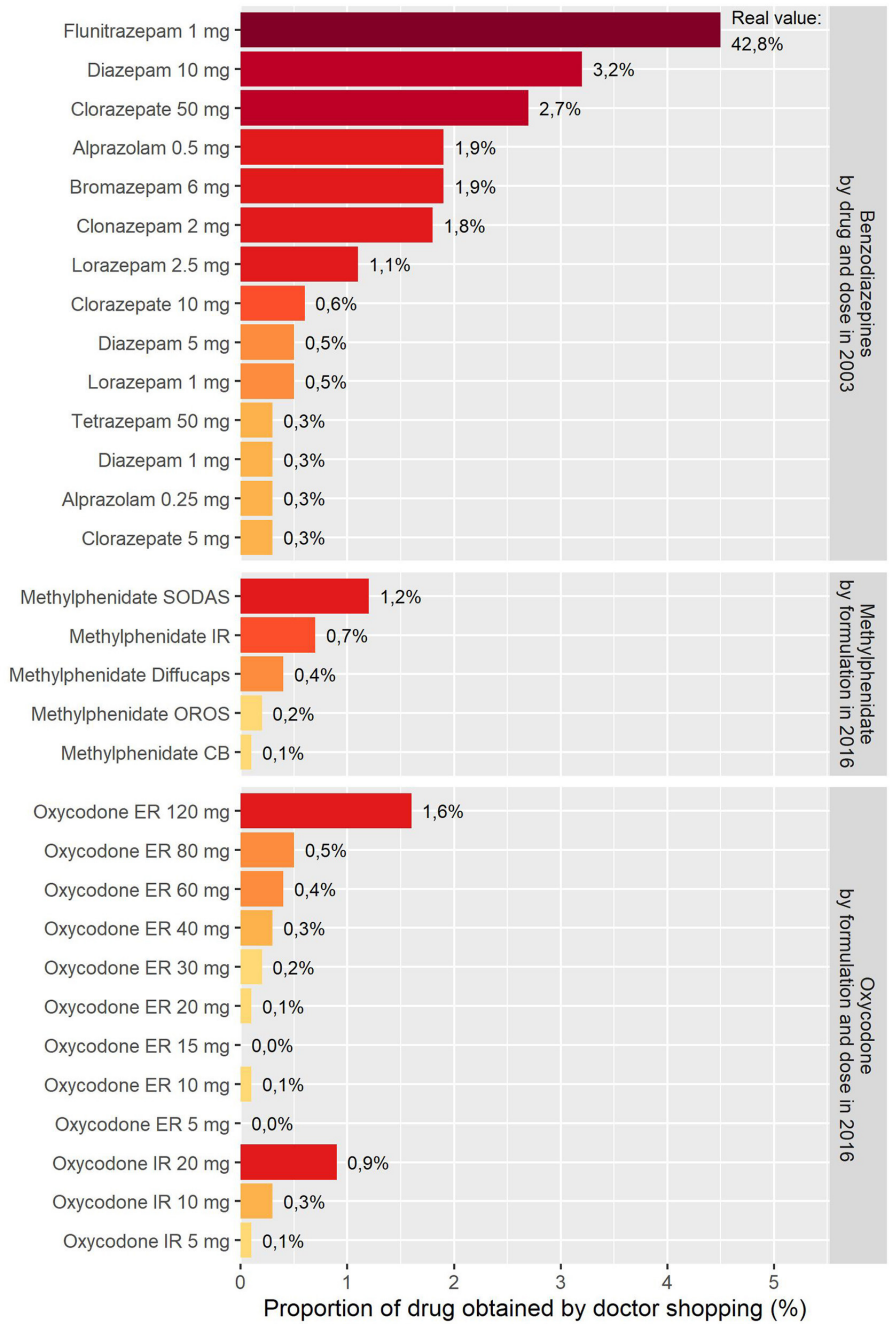
Validation of doctor shopping as a pharmacological tool through its ability to rank prescription drugs within a pharmacological class known for abuse (e.g., benzodiazepines) and recover pharmacological determinants of abuse (e.g., formulation for methylphenidate and dose for oxycodone). SODAS: Spheroidal Oral Drug Absorption System; IR: Immediate-release; OROS: Osmotic-Controlled Release Oral Delivery System; CB: Coated beads. See Table 1 in Soeiro et al. (45) for details on formulations.

The method demonstrated its ability to rank prescription drugs according to their abuse potential in the real-life setting. A study was conducted among the 128,230 patients who received benzodiazepine in a population of one million inhabitants in South West France in 2003. The method found a much higher proportion obtained by doctor shopping for flunitrazepam 1 mg (i.e., 42.8% of the quantity dispensed), then for diazepam 10 mg (i.e., 3.2% of the quantity dispensed), and clorazepate 50 mg (i.e., 2.7% of the quantity dispensed) (40) (Figure 3).

Interestingly, although flunitrazepam has pharmacological characteristics prone to abuse [e.g., rapid onset of action, liposolubility, and additive effects with alcohol (67, 68)], there is no evidence of any important experimental difference for its abuse potential compared to other benzodiazepines (69). Nevertheless, a review of the literature found that the two benzodiazepines with the highest abuse potential are flunitrazepam and diazepam (70), particularly in opioid-abusing patients, many of whom reported a preference for flunitrazepam over other benzodiazepines (69).

### Discriminating Prescription Drugs Within a Pharmacological Class Not Known for Abuse

The method also demonstrated its ability to discriminate prescription drugs by specifically detecting tianeptine among antidepressants (41). Back then, tianeptine was thought to have no abuse potential, as mentioned in the French summary of product characteristics before 2005, because there was no evidence of such a risk on the data available before approval (71). Nevertheless, the first reports of abuse with tianeptine emerged in the literature (72–75).

A study was conducted among the 410,525 patients who received an antidepressant in a population of five million inhabitants in South East France in 2005. Tianeptine ranked first among the antidepressants for the proportion obtained by doctor shopping (i.e., 2.0% of the quantity dispensed), and was close to benzodiazepines with a well-known abuse potential in the real-life setting (41). In addition to reports from other data sources, these findings led to a stricter regulation of tianeptine in France.

Interestingly, tianeptine is a selective serotonin reuptake enhancer and an opioid agonist (76), with a chemical structure close to amineptine, which was withdrawn in several countries because of the abuse associated with hepatitis (77, 78). In addition, psychostimulant effects of tianeptine appear at high doses (75). These pharmacological properties makes tianeptine an atypical antidepressant, and may account for its abuse potential.

### Recovering Pharmacological Determinants of Abuse

The method finally demonstrated its ability to recover pharmacological determinants of abuse, such as a preference for specific formulations and high doses for several pharmacological classes (e.g., benzodiazepines, opioids, and methylphenidate) (40, 42–45).

The effect of formulation is especially notable for methylphenidate, which was available in five formulations, using different extended-release technologies and ratio of immediate-release/extended-release methylphenidate in France in 2016. On the same year, a study was conducted among the 63,739 patients who received methylphenidate in the 67 million inhabitants in France. Patients with doctor shopping behavior preferred formulations with a higher ratio of immediate-release/extended-release methylphenidate (e.g., methylphenidate with Spheroidal Oral Drug Absorption System and methylphenidate immediate-release) over methylphenidate with Osmotic-Controlled Release Oral Delivery System (OROS) (45) (Figure 3). Given that the use of intravenous route for methylphenidate is frequent in France (79–81), this pattern also suggests that a part of methylphenidate obtained by doctor shopping may be used by intravenous route, because methylphenidate OROS is the least preferred drug for intravenous route in drug-abusing patients (82). Interestingly, OROS increases the time for preparing due to the viscosity of the preparation, which may be the reason for this preference (83).

Similarly, the effect of dose is especially notable for oxycodone, which was available in 12 doses from 5 to 120 mg in France in 2016. A study was conducted in 2016 among the 212,753 patients who received oxycodone in the 67 million inhabitants in France. There was a dose-response-like relationship between dose and doctor shopping (i.e., the quantity obtained by doctor shopping increased with the dose for both immediate- and extended-release tablets) (44) (Figure 3). Interestingly, as soon as 2008, the method detected a first signal for oxycodone, particularly in one region (81), although no oxycodone abuse had been detected in France back then. This finding underlines the usefulness of local monitoring to assess the geographical specificities of abuse, which may help to target public health interventions (84).

## Added Value of Doctor Shopping Monitoring to Improve the Early Detection of Prescription Drug Abuse Within a Multidimensional Monitoring

In order to face the challenges of monitoring prescription drug abuse, several authors and health authorities advocate for a multidimensional proactive post-marketing monitoring (10–12). Such a multidimensional monitoring is already operational in France through the French Addictovigilance Network (85–90). In addition to a spontaneous notification by health professionals and pharmacoepidemiological studies from claims databases (91, 92), multiple *ad hoc* studies have been conducted nationwide, such as: the OSIAP program, to detect forged prescription (93, 94); the OPPIDUM program, to detect psychoactive drug use in drug-abusing patients (95, 96); the DRAMES program, to detect deaths related to psychoactive drugs; the DTA program, to detect deaths related to analgesic prescription drugs; or the chemical submission program, to detect psychoactive drugs administered without the victim's knowledge (97).

**Figure 4:**
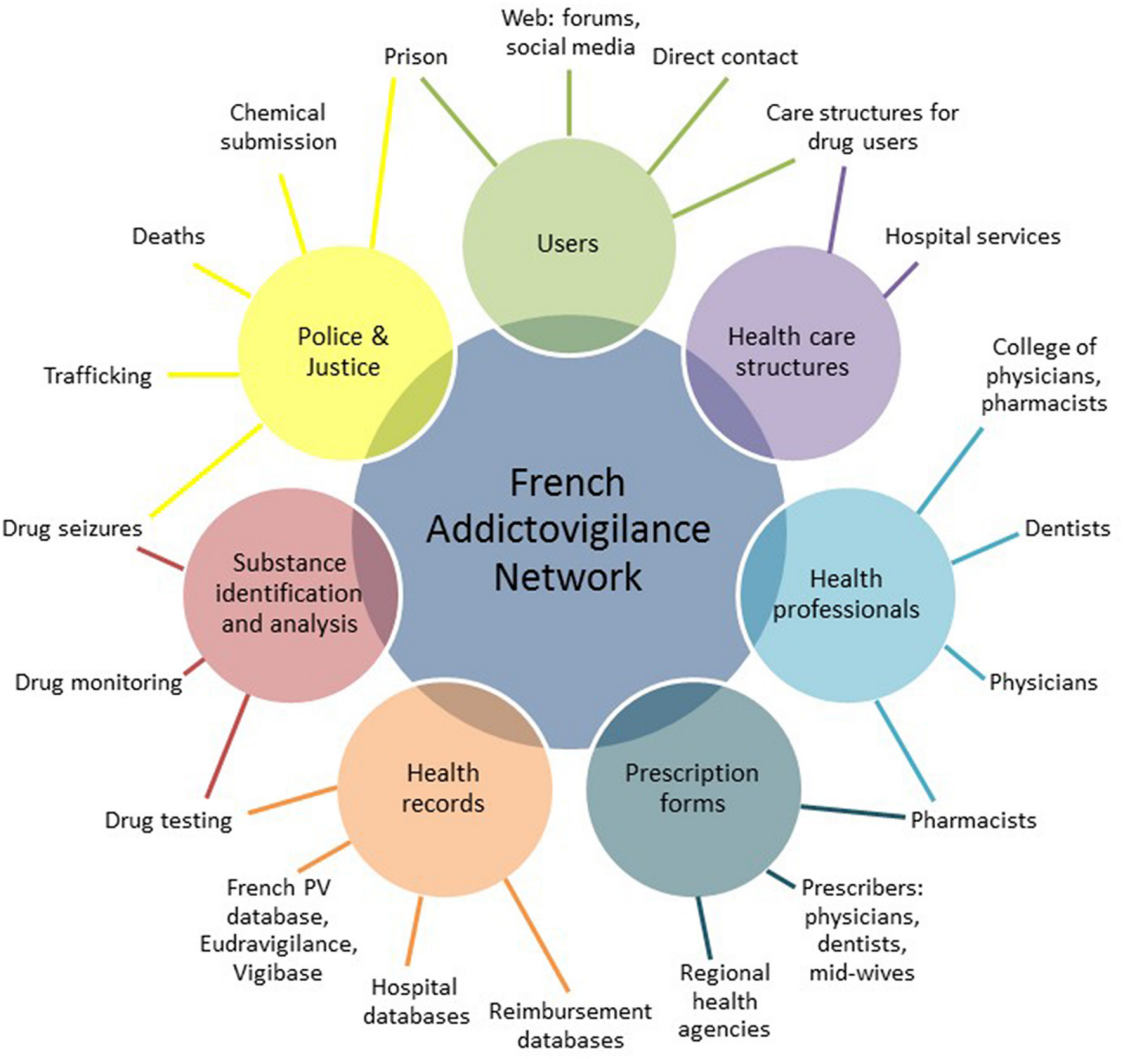
Multidimensional monitoring conducted by the French Addictovigilance Network to detect signals by crossing complementary data sources.

The multidimensional monitoring conducted by the French Addictovigilance Network enables the detection of signals by crossing complementary data sources, which overcomes the limitation of each data source taken individually (Figure 4). In addition to the already existing programs of the French Addictovigilance Network, the added value of doctor shopping monitoring is its ability to exhaustively detect drug-abusing patients in the general population, and for all the marketed prescription drugs. This ability in not only theoretical, as demonstrated by a nationwide quantification of doctor shopping recently conducted in France for 220 psychoactive prescription drugs from many pharmacological classes (e.g., opioids, benzodiazepines, stimulants, antihistamines, gabapentinoids, antidepressants, and antipsychotics) (98). Given its automatic nature, the method can be implemented routinely, with minimal costs and limited workforce. Interestingly, doctor shopping monitoring is not impaired by under-declaration. Such features make doctor shopping monitoring a complementary tool, which is even more topical in the big-data era to assess prescription drug abuse and detect emerging trends in the field of addictovigilance as early as possible (99).

For example, the monitoring of tramadol conducted by the French Addictovigilance Network detected an increasing abuse (100, 101). Beside a regular increase in spontaneous reports, tramadol has been used in combination or in alternation with other opioids in drug-abusing patients according to the OPPIDUM program; has ranked first among analgesics for deaths in the DTA program; and has increased for falsified prescriptions in OSIAP. These converging data are further strengthened and complemented by the nationwide quantification of doctor shopping in France (98). Notably, tramadol ranked ninth among 220 psychoactive prescription drugs for the quantity obtained by doctor shopping (i.e., 755,333 DDD). From 2010 to 2016, tramadol was one of the few opioids for which both quantity and proportion obtained by doctor shopping increased (i.e., +12% and +5%, respectively). In the population approach, tramadol ranked first for the number of patients with doctor shopping behavior (i.e., 44,088 patients). Interestingly, tramadol is an atypical opioid analgesic that also inhibits the reuptake of serotonin and norepinephrine (102). In addition, O-desmethyltramadol, which is produced by the polymorphic cytochrome P450 2D6, has a 200 to 500 higher affinity for μ-opioid receptor than tramadol (103). In light of the pharmacological properties of tramadol and international data (104–106), these increasing trends are strong signals in the French context.

## Discussion

The paper aims to review how doctor shopping monitoring can improve the early detection of prescription drug abuse within a multidimensional monitoring. The paper provides an in-depth overview of two decades of development and validation of the method as a complementary component of the multidimensional monitoring conducted by the French Addictovigilance Network. In this context, doctor shopping monitoring has demonstrated its added value to improve the early detection of prescription drug abuse. Notably, the monitoring must also include a strong pharmacological expertise, which is essential to both analyze signals and interpret pharmacoepidemiological data.

While the method has been developed and validated in France, the rationale is transposable in other health systems with available claims databases. In practice, given the increasing availability of claims databases in several countries, the main issue is to integrate doctor shopping monitoring within a multidimensional monitoring. In addition, the method must undergo an in-depth customized validation process, accounting for the specificities of the targeted health system (e.g., availability of prescription drugs and illicit alternatives, cost of prescription drugs and visits, prescription and control methods, and risks involved for fraud).

Such pharmacoepidemiological monitoring is intended to develop in the big-data era. Interestingly, it is nowadays technically possible to implement a real-time doctor shopping monitoring, assuming that a quick access to data is available, which is currently the bottleneck.

Finally, as a public health mission, monitoring prescription drug abuse must rely on free from conflict-of-interest organizations to prevent private interest from interfering, as it was the case in the opioid crisis (107, 108). This is even more necessary given that such monitoring may lead to the reconsideration of the safety of some prescription drugs in the real-life setting, and trigger regulatory measures. Among them, prescription monitoring programs are efficient to mitigate doctor shopping and its consequences (39, 109, 110). Nevertheless, the consequences of such regulatory measures must be globally assessed, because hardening the access to prescription drugs may lead to switching to illicit drugs. The challenge is to develop methods that maximize the detection and prevention of prescription drug abuse, while minimizing any adverse impact on legitimate medical treatments.

## Conclusion

To conclude, doctor shopping monitoring is a useful component for an efficient multidimensional monitoring to improve the early detection of prescription drug abuse in the field of addictovigilance.

## Author Contributions

TS wrote the manuscript. CL, VP, ML-M, and JM reviewed the manuscript. All authors contributed to the article and approved the submitted version.

## Conflict of Interest

The authors declare that the research was conducted in the absence of any commercial or financial relationships that could be construed as a potential conflict of interest.
